# Operationalizing “One Health” as “One Digital Health” Through a Global Framework That Emphasizes Fair and Equitable Sharing of Benefits From the Use of Artificial Intelligence and Related Digital Technologies

**DOI:** 10.3389/fpubh.2022.768977

**Published:** 2022-05-03

**Authors:** Calvin Wai-Loon Ho

**Affiliations:** Department of Law and Centre for Medical Ethics and Law, The University of Hong Kong, Pokfulam, Hong Kong SAR, China

**Keywords:** FAIR, Convention on Biological Diversity, digital, artificial intelligence, One Health, benefit sharing, International Health Regulations, data sharing

## Abstract

The operationalization of One Health (OH) through digitalization is a means to deploy digital technologies (including Artificial Intelligence (AI), big data and related digital technologies) to better capacitate us to deal with growing climate exigency and related threats to human, animal and plant health. With reference to the concept of One Digital Health (ODH), this paper considers how digital capabilities can help to overcome ‘operational brakes’ in OH through new and deeper insights, better predictions, and more targeted or precise preventive strategies and public health countermeasures. However, the data landscape is fragmented and access to certain types of data is increasingly restrictive as individuals, communities and countries seek to assert greater control over data taken from them. This paper proposes for a dedicated global ODH framework—centered on fairness and equity—to be established to promote data-sharing across all the key knowledge domains of OH and to devise data-driven solutions to challenges in the human-animal-ecosystems interface. It first considers the data landscape in relation to: (1) Human and population health; (2) Pathogens; (3) Animal and plant health; and (4) Ecosystems and biodiversity. The complexification from the application of advance genetic sequencing technology is then considered, with focus on current debates over whether certain types of data like digital (genetic) sequencing information (DSI) should remain openly and freely accessible. The proposed ODH framework must augment the existing access and benefit sharing (ABS) framework currently prescribed under the Nagoya Protocol to the Convention on Biological Diversity (CBD) in at least three different ways. First, the ODH framework should apply to all genetic resources and data, including DSI, whether from humans or non-humans. Second, the FAIRER principles should be implemented, with focus on fair and equitable benefit-sharing. Third, the ODH framework should adopt multilateral approaches to data sharing (such as through federated data systems) and to ABS. By operationalizing OH as ODH, we are more likely to be able to protect and restore natural habitats, secure the health and well-being of all living things, and thereby realize the goals set out in the post-2020 Global Biodiversity Framework under the CBD.

## Introduction

The “Code Red” warning of the United Nation's Intergovernmental Panel on Climate Change is an unequivocal message that unless drastic changes are made to a range of human activities, escalating global temperature will exert a heavy toll on biodiversity, human and animal health, and geopolitics, in quite conceivably detrimental ways (1). The rapid loss of Arctic sea ice observed in 2007 is closely matched by a parallel upward trend in the frequency and number of new diseases, where recent global transmission of infectious diseases, like Nipah Virus (1998; 2001–2018), Avian Flu H5N1 (2006–2008), SARS (2002–2003), MERS (2012; 2015; 2018), Ebola (2014–2016; 2018–2020), and Swine Flu (2009), are either known or likely to have been of zoonotic origin (2). Where the current COVID-19 pandemic is concerned, a large body of virologic, epidemiologic, veterinary, and ecologic data points strongly to the new virus, SARS-CoV-2, as having evolved directly or indirectly from a β-coronavirus in the sarbecovirus (SARS-like virus) group that naturally infects bats and pangolins in East Asia (3). With ever growing encroachment on natural habitats through deforestation and urbanization, loss of biodiversity and overharvesting, pollution, human overpopulation and climate change, the frequency of zoonosis and their economic impact are expected to grow.

Even with these impending perils on health and environment, the cohesiveness and comprehensiveness of collective action in global response have been obscure in comparison. The Sustainable Development Goals (SDGs) are similarly diffused, even if health and environment are explicit concerns. As a matter of political accountability, Lawrence Gostin and Eric Friedman highlight the importance of high-quality data and for every country to establish or upgrade health information systems (4). A robust health data infrastructure can in turn power up new digital capabilities in Artificial Intelligence (AI), big data analytics, and related digital technologies, in monitoring and reviewing health and health-related targets and concerns, as well as support decision-making across different levels (see Table 1 for key definitions). This hope is reflected in Resolution WHA71.7 of the World Health Assembly adopted in 2018 for a global strategy on digital health to be devised with the goal of supporting national health systems to meet their SDG commitments (12). Currently, the global health informatics landscape is checkered, where some health systems possess only rudimentary digital health capabilities (13). Looking beyond human health to related domains of animal health and ecosystems, the digital landscape is similarly jarring. Still, the enormous gains that data and digital capabilities offer in addressing challenges in each of the core One Health (OH) domains, and in the human-animal-ecosystem interface, is already well-recognized. Where human health is concerned, Eric Topol has famously argued that AI will help make healthcare more humane by freeing up time for clinicians, improving medical diagnosis and treatment, lowering healthcare cost and reducing human mortality (14). Facial recognition AI technology has also been applied to protect grape crops (15), and to monitor animal health in livestock farming (16). In conservation biology, AI is being deployed to analyze huge troves of image and audio data (17). Similar proposals are being made in relation to the human-animal-ecosystem interface (18), including a “One Digital Health” (ODH) framework to focus on the digital in (and the digitalization of) OH (5). These various developments, proposals and initiatives all point unmistakably to AI and related digital tools as an opportunity to turn the tide of an impending global health and environmental disaster, or to at least mitigate its impact. Digital technologies are expected to redefine the way that data is collected, manipulated and interpreted across a broad range of surveillance activities, including monitoring of earth's natural systems (e.g., for land use changes) and detection of disease pathogens. The ability to integrate and evaluate data from a broad spectrum of knowledge domains and applications could provide new insights on how existing challenges are understood, and potential solutions or pathways that could be adopted (19). From the standpoint of OH operationalization, digital technologies can better enable us to work across the varied knowledge domains of biodiversity and health to discover causal linkages, identify hidden or non-obvious associations, make predictions and guide decision-making across many levels.

**Table 1:** Key definitions (5–11).

**Access and benefit sharing (ABS)**: Refers to ways of accessing genetic resources and the fair and equitable sharing of the benefits between those who provide the resources and those who use them. **Algorithm**: A step by step mathematical methods of solving a problem, commonly used in data processing, calculation and other related computer and mathematical operations. **Application (or App)**: Computer software or program, most commonly a small, specific one used for mobile devices. **Application Programming Interface (API)**: An application that enables data to be retrieved from many types of databases and applications, including those at remote locations. **Artificial Intelligence**: The simulation of human intelligence processes by machines, especially computer systems. These processes include learning (the acquisition of information and rules for using the information), reasoning (using rules to reach approximate or definite conclusions) and self-correction. **Convention on Biological Diversity (CBD)**: An international, legally-binding treaty that supports conservation, sustainable use of biodiversity, and fair and equitable benefits arising from the use of biodiversity resources. **Deep Learning**: Subfield of representation learning that relies on artificial neural networks with multiple processing layers to learn representations of data with multiple levels of abstraction. **Digital sequence information (DSI)**: Data that are derived from the sequencing and analysis of genetic information in cells. **Federated data system**: A data architecture that uses multiple interconnected nodes, enabled by application programming interfaces (APIs), to provide secure yet open access to geographically disparate data systems and data formats. **International Health Regulations (IHR)**: An international, legally-binding treaty with purpose and scope stated as “to prevent, protect against, control and provide a public health response to the international spread of disease in ways that are commensurate with and restricted to public health risks, and which avoid unnecessary interference with international traffic and trade.” **Internet-of-Things**: Envisions a self-configuring, adaptive, complex network that interconnects “things” to the Internet through the use of standard communication protocols. The interconnected “things” have physical or virtual representation in the digital world, sensing/actuating capability, a programmablility feature and are uniquely identifiable. The representation contains information including the thing's identify, status, location or any other business, social or privately relevant information. For a low complexity system, an Internet-of-Things (IoT) system is a network that connects uniquely identifiable “Things” to the Internet. The IoT system can grow to a level of complexity where a large amount of “Things” can be interconnected to deliver complex service and support an execution of a complex process. **Machine Learning**: An application of AI that provides systems with the ability to automatically learn and improve from experience without being explicitly programmed. Machine learning focuses on the development of computer programs that can access data and use it to learn for themselves. **Model**: Structure and state of a neural network that allows the transformation of input data into a calculated output. **Mutually agreed terms (MAT)**: The terms of use negotiated between the user and provider of a genetic resource. **Nagoya Protocol (NP)**: An international agreement aimed at sharing the benefits arising from the use of genetic resources in a fair and equitable way. **Neural Networks**: A series of algorithms that endeavours to recognize underlying relationships in a set of data through a process that mimics the way the human brain operates. **One Digital Health (ODH)**: A novel framework that aims to facilitate and improve collaboration among practitioners of One Health and digital health communities. **One Health (OH)**: One Health is a collaborative, multisectoral, and trans-disciplinary approach—working at local, regional, national, and global levels—to achieve optimal health and well-being outcomes recognizing the interconnections between people, animals, plants and their shared environment. **Prior informed consent (PIC)**: The informed agreement or consent of a competent national authority of the provider country given to a user, and arrived at before access to genetic resources for the exchange of resources or products. **Sensor**: A device identifying or recording features of a given physical entity **Testing**: Process of evaluating the performance of a model. **Thing**: Generally speaking, any physical object. In the term “Internet-of-Things,” however, it denotes the same concept as a physical entity. **Training**: Process of selecting the ideal parameters of a model after iterative adjustments. **Validation**: Process of using a subset of the dataset (distinct from the training set) to adjust the parameters of a model. **Whole genome sequencing (WGS)**: the process of determining the entire DNA sequence that makes up and organism.

In fact, data has been a central feature of OH, with a key concern being the actual and potential movement of diseases among humans, domestic animals and wildlife populations, notably in this principle (20): “Devise adaptive, holistic and forward-looking approaches to the prevention, surveillance, monitoring, control and mitigation of emerging and resurging diseases that take the complex interconnections among species into full account.” Data remains central to the current conception of OH, as a global strategy that aims to, among other goals, evaluate health risks presented by the emergence or re-emergence of infectious or non-infectious diseases through a holistic and transdisciplinary approach that gives emphasis to the human-animal-ecosystem dynamics of global changes brought on by population growth, industrialization and geopolitical challenges (21). Data is also central to a tripartite collaboration set up by the World Health Organization (WHO), the World Organisation for Animal Health (OIE), and the Food and Agriculture Organization of the United Nations (FAO) to address concerns that arise from the human-animal-ecosystem interface. Each of these international organizations support countries in building capacity for indicator and event-based surveillance, with communication channels set up pursuant to the OIE's Terrestrial and Aquatic Animal Health Codes (22, 23), the WHO's International Health Regulations (IHR) (6), and the Codex Alimentarius Commission guidelines of the FAO and the WHO (24). As a collaborative initiative, the Global Early Warning System for Major Animal Diseases Including Zoonosis (GLEWS) was established in 2006, in response to health threats like H5N1 highly pathogenic avian influenza and SARS (25). In 2013, GLEWS was upgraded as GLEWS+ to provide a cross-sectoral mechanism for conducting joint risk assessments in order to formulate risk management options for health events at the human-animal-ecosystems interface. GLEWS+ is linked to networks like the International Good Safety Authorities Network (INFOSAN) and the joint OIE/FAO Network of Expertise on Animal Influenza (OFFLU) (26). A recent application of the GLEWS+ was directed at assessing risks relating to the introduction and spread of SARS-CoV-2 within mink fur farms, and spillover of the virus from the farms to humans and susceptible wildlife (27).

Even if data (whether as “the new oil” or “new nuclear power”) (28) is available, it is dispersed across multiple sources in highly heterogeneous databases, and there are technical (e.g., lack of metadata or schema for data identification and consolidation), as well as ethical and legal, barriers to access and use. Additionally, data will need to be properly curated before it could be applied to AI and related digital technologies. Ideally, data repositories should be transparent about their databases and managed in a manner that is consistent with the FAIR principles (i.e., Findable, Accessible, Interoperable and Reusable) (29). Ironically, many difficulties to operationalizing these principles arise from the lack of fairness that persists across all the three key domains (i.e., human, animal and ecosystem) of OH. Individuals do not want to share their health data for fear of stigma, unfair discrimination and exploitation. Researchers, particularly those in the Global South, still do not receive adequate recognition—and may even be penalized—for sharing data or otherwise enabling this. Countries are similarly unwilling to share biological materials (including pathogens) and data unless there is assurance of fair access to benefits (e.g., vaccines) that may directly or indirectly arise. In other words, the FAIR principles are unlikely to promote data sharing unless and until more fundamental and deep-seated inequities are addressed. For this reason, it has been proposed that “Ethical” (and “Reproducible”) be added to the existing FAIR principles, as FAIRER principle are more consistent with Open Science (30).

Globally, the Convention on Biological Diversity (CBD) (31), and the Nagoya Protocol (NP), are international agreements that have attempted to address inequities that could arise from the utilization of genetic resources through the establishment of an international law framework on access and benefit sharing (ABS) (32). As there is no international treaty or convention that is specific to OH, the ABS framework has been foundational to the access and management of non-human genetic materials, but neither the CBD nor the NP applies to human genetic resources (33). More recently, there have been calls for certain data, particularly “digital sequence information” (DSI) derived through the application of high-throughput genetic sequencing technology to biological organisms, to be excluded from the ABS framework in order to avoid burdening OH-related endeavors and scientific research, more generally. Where the sharing of health-related data is concerned, the IHR has been the main normative instrument of the WHO in sustaining different arrangements, frameworks and initiatives for ongoing surveillance of infectious diseases, timely risk assessment, implementation of public health control measures and access to medical interventions. For instance, the Global Health Observatory (GHO) (34), the Pandemic Influenza Preparedness (PIP) Framework (35), and the Global Action Plan (GAP) on Antimicrobial Resistance (AMR) (36), are developed under the auspices of the WHO in response to a range of global health security threats. Even though there are shared ethical commitments to securing fairness and equity, and to the protection of global public health, in the CBD, the NP and the IHR, there is no explicit connection between the CBD and NP on the one hand, and the IHR on the other, particularly where issues of OH or ODH are concerned. As we shall discuss below, this has led to legal uncertainties over international ABS arrangements, such as the sharing of certain pathogens under the PIP Framework.

With reference to the current controversy over the possibility of monetizing benefit sharing by restricting access to DSI and similar data, it is argued in this paper that the mere exclusion of such data from the ABS framework will not promote data sharing or otherwise enhance OH operationalization. The shift in bioprospecting from testing of natural products to screening of chemical libraries, and challenges that this presents to ABS and related difficulties in traceability and enforceability, have already led a number of countries to introduce laws that restrict access to biological materials and related data within their jurisdictions. Instead, operationalizing OH as ODH to enable more effective use of data and data processing tools like AI and related digital technologies will require fairness and equity to be prioritized. It is further argued that fairness and equity should be anchoring principles of a dedicated international framework that systematizes ABS requirements for all biological materials and related data, whether of human or non-human origin. This dedicated framework (say, “ABS+ framework” for ease of reference) should apply the FAIRER principles, with the “E” being clearly inclusive of fairness and equity, and possibly with equity being prioritized over other relevant ethical principles as the context requires. Additionally, the ABS+ framework will need to engage explicitly with digitalization, thereby re-orienting the proposed ODH toward building digital capacity through means that include equitably linking up databases and data platforms in order to integrating biodiversity and health in operationalizing OH. Essentializing the “digital” may help to highlight the risk that digitalization could also exacerbate inequity in, for instance, an increasingly conspicuous digital divide owing to different pace of digitalization, data proliferation and fragmentation, and the uneven distribution of digital tools, resources, knowledge and skills. Whether as a standalone arrangement or otherwise as part of an international treaty, the ABS+ framework should bring together people and technology, and to sets out clear ethical and legal objectives in order to initiate change in cultural attitude, as well as social and institutional practices, and to overcome the value-action gap (37). Additionally, a different mindset is needed to ensure that the use of data should not be compliance driven (as it appears to be the case for the ABS framework and IHR), but should nurture a culture of learning, participation and good governance.

In conceptualizing the ABS+ framework as a crucial component of ODH, the methodology of normative conceptual analysis is applied. As Kenneth Einar Himma explains, traditional conceptual analysis attempts to construct a narrative about something (e.g., fair and equitable ABS) that comes under the concept (e.g., OH) based on general intuitions or understanding (38). For example, conceptual analysis of law is concerned with identifying those properties that need to be present in order for something to be understood as a legal phenomenon (as opposed to one that is scientific or social). Traditional conceptual analysis is not concerned with causation and tends to be purely descriptive. In contrast, normative conceptual analysis draws on ethical or moral norms to explain what the concept should be (rather than only as what it is). For instance, jurisprudence or legal theory is usually concerned with systematizing law, critically analyzing its workings and identifying its failings and potential, in order to make it operate in more meaningful or valuable ways (39). One such methodological approach will require the theorist to attend to the good reasons that humans have to adopt and maintain a legal system (40). Where health-related jurisprudence is concerned, moral or ethical theories tend to be relied on, along with (at a minimum) a sensitivity to empirical sociolegal inquiries (41). As a concept, ODH is explained in this paper in terms of digitalization in the four key knowledge domains in order to devise data-driven solutions to challenges in the human-animal-ecosystems interface. While this aspect of the paper is essentially descriptive, the argument that the ABS+ framework needs to be an integral part of ODH is normative. In other words, the concept of ODH goes beyond being a descriptive account of the digitalization of OH in the four knowledge domains, by also considering the moral value of its content, operationalization and practices in fair and equitable ABS, since the two enterprises (i.e., descriptive and normative) cannot be meaningfully separated. However, metaphysical inquiry is beyond the scope of this paper, although it should be noted that the explication of a concept may include generalizable claims (or “truths”) that go beyond the conventions that establish the core meanings of the concept.

In the section that follows, we first consider some means by which AI and other digital capabilities can support the operationalization of OH as ODH. We also consider the “operational brakes,” particularly in the uneven uptake of AI and related digital technologies (being higher in the human health domain rather than in animal health) and the difficulties in locating and integrating heterogeneous and dispersed data sources. The latter is an obstacle to ODH as the development and effective deployment of AI and related digital technologies are contingent upon the availability of data that is of adequate quality. Following this, the data landscapes on human, animal and plant health, on pathogens and on ecosystems and biodiversity are broadly discussed from a OH standpoint to highlight the lack of a unifying framework as possibly a key contributor to data gaps, fragmentation and dispersion. While the collection and sharing of disease surveillance data has operated relatively smoothly under the IHR, the same cannot be said of pathogens and genetic data or DSI. Previous contentions over the sharing of the H5N1 pathogen and current debates over the status of the DSI in relation to the ABS framework bring fair and equitable sharing of benefits to the fore as a central concern to operationalizing OH as ODH. The paper concludes with an argument for the establishment of an ABS+ framework that applies to both human and non-human genetic materials and related information, and sets out some features that this single and comprehensive framework should include.

## Artificial Intelligence and One Health Digitalization

The OH strategy has led to better understand of the emergence or re-emergence of diseases in at least three areas (21): (1) How particular uses of land (e.g., agriculture) contributed to disease transmission, so that better land use could minimize exposure to pathogens (42, 43); (2) How a disease outbreak (e.g., outbreaks of Schistosomiasis in Europe in 2013 and 2015) may be attributable to multiple contributors (e.g., presence of intermediate host, absence of ruminant reservoir hosts, and improved diagnostic tools); and (3) How modeling diseases in social networks could help to predict outbreaks and develop strategies to avoid epidemics and epizootics. In the context of public health, modeling the spread of an epidemic enables the implementation of more targeted public health intervention (whether through vaccination or other public health measures) to break the chain of transmission. This is done through identifying the risk of infection and infecting conspecifics based on the position of the individual within the network, and also the shape of the network, expressed in concepts of efficiency, resilience and nestedness. Ecological pressures, as well as intra- and interspecific relationships may be integrated into these models, which could in turn lead to better understanding and/or insights into ecological buffers that could be deployed to ensure robustness or resilience (44).

In more recent years, the growth of data in genomics and metagenomics, along with a greater diversity of information (including analytical ones like methylation maps), and advancements in cloud and supercomputing processing technologies, have contributed to the application of AI concepts and methods for large scale studies. Important inroads have been made to harness enhanced computational power and the use of statistical methods, notably Bayesian methods, to better understand diversity and complexity in animal epidemiological systems and socio-ecological systems in OH (e.g., large scale, multi-host and multi-pathogen systems) (45). High-speed telecommunications, Internet-of-Things, location trackers (like GPS) and sensors present the opportunity to better understand the health effects of urbanization, movement and social networks (46). The social network to which a person belongs to could provide information on the likelihood of exposure to a pathogen or being affected by hypertension or obesity (47). Here too, statistical methods and multi-agent simulation have been applied to identify how urban environment and social practices impact health and well-being, and to inform urban planning and development, and a variety of public health interventions (48).

AI and big data technologies can enable us to better understand and address complex challenges in OH, where challenges could involve a wide range of knowledge systems, conditions and data types, multiple species interactions and networks across different spatial and temporal dimensions, alongside varied and possibly conflicting interests and expectations. There is now enhanced capability to collect and process data in real time through the use of sensors and advance analytics for humans and for animals (49–51). This would in turn contribute to early detection of infection, allow early intervention and support rationalization of treatments. Additionally, concerns with human and animal health is encapsulated not only in OH, but related concepts of EcoHealth and Planetary Health, all of which seek a more holistic, multidisciplinary and participatory approaches to complex health-related issues, including multi-host pathogen transmission and the impact of climatic changes on disease patterns (52). Digital modalities could enable “adaptive monitoring” to assess exposure and organism response on both an individual level and at the population level (53, 54). This could also be done across spatial and temporal scales that involve a range of samples, biomarkers and end-points. Digital tools enable integration across different multiscape approaches to study how a population of humans or animals responds to multiple stress factors in terms of their genetic and epigenetic capacities to adapt to stress factors (55).

To help control pathogen vectors (e.g., mosquitoes) or parasites, AI-based machine learning has been applied to identify genotypes of animal pathogens that are more likely to infect humans through classification of potential reservoirs of zoonotic diseases (56). Deep learning AI has also been deployed to identify the level of genetic introgression between wild and domesticated animal populations to better understand pathogen transmission in complex system networks (57). On the AMR front, AI-based software has the potential to use genetic sequencing information to predict whether a microorganism can produce antibiotics or has developed resistance against antibiotics (58). Where a microorganism has the potential to produce antibiotics, the relevant genetic information from that microorganism may be used to produce the desired antibiotics. In the context of the current COVID-19 pandemic, data sharing has supported the development of vaccines and diagnostic tests (59). Digital means of tracing and monitoring the COVID-19 pandemic have also been important in the identification of new variants of SARS-CoV-2 (60). However, AI concepts and methods (e.g., multi-agent systems) have been rarely applied on real data to study animal health issues compared with human health (61), even though an increasing volume of information is being collected about animals, pathogens and their environments (62). Not unlike human health, AI-based software could: (1) evaluate animal epidemiological systems and potentially complex situation (such as an infectious disease outbreak) and the attending dynamics; (2) study the environment in terms of particular patterns, chemical forms, signals (such as those from international surveillance of epizooties, or mortality from necropsy reports and other veterinary documents), and the like; and (3) support systems for decisions that could range from diagnosis to resource allocation.

Operationalizing OH as ODH could help to address currently inadequate communication across disciplines on political and economic challenges. Delphine Destoumieux-Garzón et al. observe that sectoral partitioning among public health, agronomy policy and other sectors of activities contribute to risks of infection (21). Since the agriculture/public health interface does not come under the competence of farmers or vector control services, the risk of insect vectors developing resistance to agricultural insecticides also increases. As Arriel Benis et al. explain, ODH prsents a means of meeting this challenge as its goal is to unify the three “perspectives” of individual health and well-being, population and society and ecosystems through digital technologies that permeate them all (5). From this data science angle, ODH is based on digital health (encompassing health informatics), One Health and environmental research, and may be engaged with through any one of its five dimensions (i.e., citizens' engagement, education, environment, human and veterinary health, and healthcare in the fourth industrial revolution). While ODH explains how digital technologies could be deployed to support communication across the different domains, the highly fragmented, distributed and heterogeneous data landscape remains a fundamental obstacle. As Destoumieux-Garzón et al. have also observed, the absence of policies that supports the collection, integration and use of a wide range of data (e.g., multiannual demographic variations of organisms, landscape changes, practice changes, rearrangement of communities in response to these factors etc.) to support analyses across different spatial and temporal scales is another OH “operational brake” (21).

In the sections the follows, we broadly consider the data landscapes in relation to: (1) Human and population health; (2) Pathogens; (3) Animal and plant health; and (4) Ecosystems and biodiversity. The complexification from the application of advance genetic sequencing technology and growing awareness of rights and interests over data on the part of individuals, communities and countries are among the emerging conditions that underscore the need to address concerns at the human-animal-ecosystem interface, particularly those that pertains to fair and equitable sharing of benefits, and to propelling OH forward as ODH, through a consistent and multilateral platform.

## Human and Population Health

There are large data sources on individual and population health that could be leveraged on for new knowledge and insights on zoonoses under the proposed ODH approach, which seeks to support rapid and more accurate methods for detecting disease trends, outbreaks, pathogens, and causes of emergence. As considered above, the GLEWS+ is an important source of disease surveillance data. Another event-based surveillance network that is supported by the WHO as part of its Health Emergencies Program is the Global Outbreak Alert and Response Network (GOARN), which comprises over 240 public health institutions, international organizations, non-governmental organizations, United Nations agencies, and also other disease specific and technical networks. Established in 2000, GOARN coordinates (collaboratively with FAO and OIE) rapid international support teams, provide assistance to countries to investigate and characterize events, assess risks, strengthen outbreak response, and support national outbreak preparedness (63). Other important infectious diseases surveillance networks include the Connecting Organization for Regional Disease Surveillance (CORDS) with goals that are aligned with those of OH (64). Additionally, there are a number of initiatives being taken up or proposed under the GAP-AMR that will be of direct relevance to the ODH approach (19). These includes a global system of DNA genome database for microbial identification and diagnostics to enable professional response to health threats in all countries with basic laboratory infrastructure or better (65).

The collection and sharing of disease surveillance data and other OH informatics in response to an actual or potential global health security threat are coordinated under the auspices of the IHR, which also designates the WHO as the international agency responsible for its implementation. The 196 State Parties to the IHR are required (under Articles 5 and 6) to assess all unusual health events occurring within their jurisdictions and to notify WHO of all events that may be a “public health emergency of international concern” (PHEIC). More generally, all State Parties are required to have or develop minimum public health capacities for the effective implementation of the IHR (34). Since 2019, a digital platform (known as e-SPAR or electronic State Parties Self-Assessment Annual Reporting Tool) has been the means by which State Parties could report across 13 IHR core capacities, which include surveillance, laboratory capacity, zoonotic events and the human-animal interface, and legislation and financing (66). The IHR does not have any explicit provision on ABS of pathogens, but such an obligation could arguably be derived from the general obligation under the IHR to cooperate in good faith, since the sharing of pathogen is essential to enable surveillance and response, the effectiveness of which may be directly linked to the characteristics of a specific pathogen (e.g., influenza) (67). ABS arrangements (if any) have historically been informal, and have operated on the assumption that a party with custody over tangible biological materials or control over data also has the right to manage its use and disposal. For this reason, the provenance of biological materials and related data in historical archives is often unclear as their collection or handling was not supported by written documentation, or otherwise pursuant to formal approval obtained from national authorities. In more recent times, individuals and communities are recognized to have control over their biological materials and health data (especially if it identifies particular individuals or communities) for a number of reasons, including the scientific and commercial value of these resources, historical abuses and exploitations that led to greater ethical awareness and expectations, and legal safeguards that have been introduced. As a general ethical requirement, human biological materials and health data should only be collected or used after the individuals to whom they relate (and sometimes their communities also) have consented to this on a voluntary and informed basis (68). However, the requirement of informed consent may be dispensed with (usually through legislation or regulation) in a limited number of circumstances and its specificity may vary by country (see for instance, Guideline 10 of the International Ethical Guidelines of the Council for International Organizations of Medical Sciences on modifications and waivers of informed consent) (69). The control that informed consent confers should not be confused with ownership, as individuals do not have a fundamental legal right to monetize and sell their biological materials (with some limited exceptions (e.g., human hair) in some countries) and health data (70). Instead, there is also a general expectation for individuals and communities to contribute their biological materials and/or health data – often altruistically—to uses that promote the common good of all, such as research and public health purposes (71, 72).

**Figure 1:**
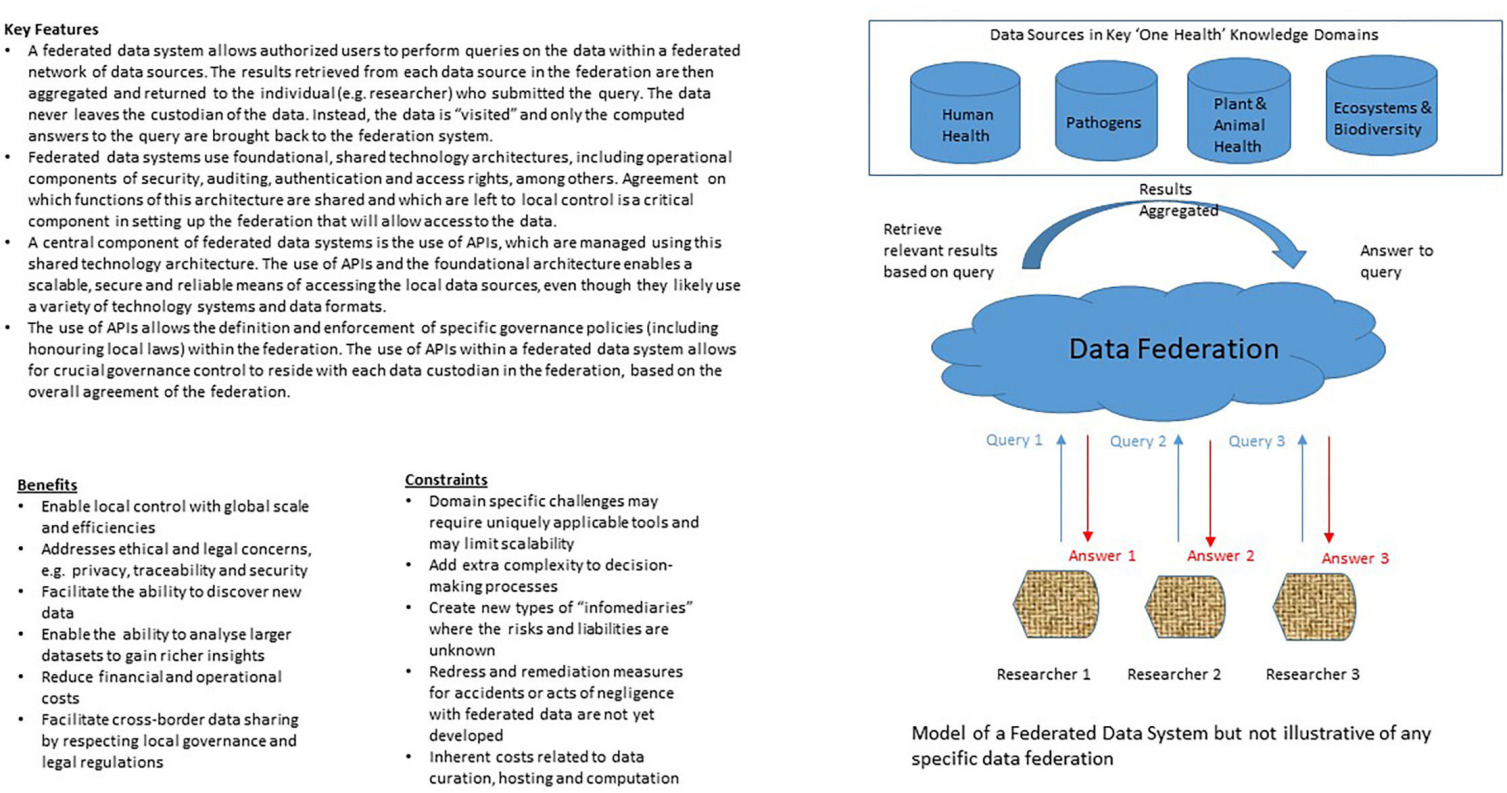
Key elements of federated data systems (8, 79, 80).

With growing interest in digital health, there will be increasing demand for high-quality health data to develop, train and validate AI devices and systems. At the same time, the technological capability of obtaining health-related data that is not only more granular (e.g., through whole genome sequencing) but also more varied (e.g., through sensors linked up through the Internet-of-Things), and the ability to process it rapidly and efficiently (e.g., through cloud and edge platforms), will also grow. These unprecedented developments are important in transforming healthcare to become more precise and personalized, and in bringing clinical care closer to public health in the application of predictive modeling and shared emphasis of disease prevention (73). However, these developments also weaken the control that individuals (and countries) have over their health data, which could have in turn contributed to data protection laws being introduced in over 100 countries (74). The extent that individuals should be able to restrict access to their health-related data or its collection and use, particularly by commercial entities or for commercial purposes, and whether they (or their communities) should derive benefit from the products, services and/or (AI) methods that are subsequently developed are key concerns in current discussions on data-sharing arrangements and governance. Unlike the special conditions that applied to the Human Genome Project about two decades ago where human genome sequences were quickly made openly accessible (75), some form of controlled-access will be an increasingly commonplace feature of human and population health databases today (76). It is as yet unclear if any arrangement that is short of open access will pose an undue burden on important research and public health activities (77), but it is perhaps interesting to note that the strongest data protection regimes tend to be found in scientifically advanced and developed countries. From a values-based perspective, equity and fairness (to be discussed below) provide justification for controlling access to human and population health data, as opposed to a data landscape that is free-for-all (78). If correct, the question to consider next is what type of controlled-access arrangement can best advance equity locally and globally? While it is beyond the scope of this paper to consider all forms of controlled-access arrangement and their equity implications, data federation (see Figure 1) appears to be a viable way forward and will be discussed below.

## Pathogens

With growing recognition of the scientific and commercial value of biological materials and related data, shared norms and rules are needed to ensure that these resources remain accessible in ways that are responsible, transparent, fair and equitable, and for purposes that advance the common good. The essence of these norms and rules are embodied in the three principles that underscore the CBD: (1) conservation of biological diversity; (2) Sustainable use of biological diversity; and (3) equitable sharing of the benefits that arise from the use of biological diversity. This treaty has been adopted by many sovereign states (known as “contracting parties”) since 1992, although the United States (US) has yet to ratify the convention. Other goals set out in the CDB includes the promotion of technical and scientific cooperation (Articles 12 and 18) and exchange of information from all public sources (Article 17). In 2010, the NP was adopted as a supplementary international agreement to implement the ABS obligations of the CBD. Under the NP, contracting parties must monitor the use of non-human biological materials and related traditional knowledge (referred to as “genetic resources,” defined in Article 2 as genetic material composed of material of plant, animal, microbial or other origin containing functional units of heredity) that originate from within their jurisdiction. They may also regulate access to genetic resources as a provider country, and on that basis, require that benefits from the utilization (whether commercial or non-commercial) of genetic resources are fairly and equitably shared with it pursuant to the ABS rules set out in the NP. The “utilization of genetic resources” refer to “conduct research and development on the genetic and/or biochemical composition of genetic resources, including through the application of biotechnology [as defined in the CBD]” (Article 2(c) of the NP). The NP reiterates the need for scientific cooperation and technological transfer as important contributions to sustainable development.

Access is typically regulated through a system of non-transferable permits, along with stipulations on commercialization of findings arising from the genetic resource. Where R&D activities are concerned, grant of access is also conditional upon a local research institution being involved. Under the ABS framework, access is subject to prior informed consent (PIC) of the provider country (which would in practice be the local CBD National Focal Point) and only on the basis of mutually agreed terms (MAT) that include fair and equitable sharing of benefits arising from the genetic resources utilized. Benefits that arise from the utilization of genetic resources could be monetary or non-monetary, and may include joint ownership of intellectual property and acknowledgment of contributions, system-level initiatives that support public health, access to affordable treatments and capacity building.

While the definition of genetic resources does not explicitly mention pathogens, Article 3 of the CBD has since been clarified to include viruses as the sovereign genetic resources of contracting parties and are subject to the ABS rules under the CBD (81). During the “Avian flu” public health emergency in 2006, Indonesia famously invoked Article 15.1 of the CBD to claim sovereign rights over the H5N1 viruses that were isolated from within its jurisdiction, and would not share the pathogen with WHO and its Global Influenza Surveillance and Response System (GISRS) without assurance of benefit sharing. Information and materials that are shared through the GISRS have been used by vaccines manufacturers to produce influenza vaccines. After several years of negotiation, the PIP Framework was adopted as a non-legally binding instrument by the World Health Assembly (WHA) in 2011 to manage (pursuant to Article 3.1 of the PIP Framework) the sharing of H5N1 and other influenza viruses with human pandemic potential (or PIP Biological Materials, as defined under Article 4.1 of the PIP Framework), as well as benefits from the development of vaccines, diagnostics and antiviral medicines. The manner of sharing under the PIP Framework is essentially characterized by two sets of contractual terms set out as standard material transfer agreements (SMTAs). The first set of contractual terms (say, Type 1 SMTA) governs the relationship between the WHO Member State providing the PIP Biological Materials and WHO, whereas the second set of contract terms (Type 2 SMTA) is concerned with the relationship between WHO and third parties, such as vaccine producers.

Strengthened by the adoption of the PIP Framework, the GISRS remains the most developed and advanced of WHO's coordinated system of laboratories. As of January 2022, the GISRS includes 148 National Influenza Centres (NICs), seven WHO Collaborating Centres (CCs), four Essential Regulatory Laboratories, and 13 H5 Reference Laboratories (82). However, there is no clear legal connection between the PIP Framework and the NP, even though both are developed in response to the “Avian flu” crisis (83). There appears to be a degree of consensus among legal scholars that the PIP Biological Materials governed under Type 1 SMTA of the PIP Framework may be regarded as a specialized international ABS instrument under Article 4.4 of the NP, and thereby fall outside the purview of the CBD or NP (the FAO's International Treaty on Plant Genetic Resources for Food and Agriculture (ITPGRFA) being another specialized international ABS instrument, to be discussed below) (83–85). However (and as noted above), the scope of the PIP Framework is extremely narrow as it applies only to influenza viruses with human pandemic potential, such as influenza H5N1, but not to seasonal influenza viruses which cause epidemics every year. Hence, ABS requirements under the CBD continue to apply to the sharing of non-influenza pathogens or those that pose “present or imminent emergencies that threaten or damage human, animal or plant health” [under Article 8(b) of the NP]. Such a situation presents a number of challenges that include (86): (1) uncertainty regarding the scope and implementation of the ABS framework, since it is unclear if, for instance, a contracting party to the CBD is required to observe its commitments under the ABS framework if it is to receive a pathogen from another jurisdiction that is not; (2) the high transaction costs of implementing a bilateral system for access and sharing, as some WHO Member States not only lack the financial means, but also the technical and legal capacity or capability implement such a system; and (3) the complexity of varying domestic ABS legislations, and lack of clarity over which government entity could grant access to the pathogens after transfer, whether intellectual property rights could be sought over the pathogens, and whether benefit-sharing could be linked to access.

In a recent study on how the implementation of the NP might affect the sharing of pathogens and the potential public health implications, the WHO Secretariat recommended improving the harmonization between the ABS framework and existing systems to share pathogens and related data. The latter includes establishing new or specialized ABS instruments under the NP, introducing implementing legislation that supports public health (especially under Article 8(b) in response to present or imminent emergencies of a national or international scale) and raising awareness for, as well as supporting, international collaboration. The creation of a code of conduct for pathogen sharing to promote access for public health purposes, particularly when use is non-commercial, was also proposed (87).

Reluctance on the part of countries to allow unrestricted access to pathogens (or genetic sequencing data thereof) through networks like the International Nucleotide Sequence Database Collaboration (INSDC), notably following the outbreak of H5N1 in 2006, led to the establishment of the Global Initiative on Sharing All Influenza Data (GISAID) in 2008 (88). While access to GISAID's database is free of charge, users must provide their identifiable information and must agree to the terms set out in the Database Access Agreement. These terms include the requirements for users: to seek permission from data providers to republish the data, acknowledge the laboratories that provided the specimens in scientific publications, to make every effort to include these laboratories in research on the sequence data, and to ensure that there is open sharing of data within GISAID. These requirements are consistent with the ABS framework as GISAID has clear records of the geographical origins (or provenance) of specimens and related data, as well as the identities of all parties involved.

Since its launch GISAID has facilitated data sharing among the WHO collaborating centers and national influenza centers for the bi-annual influenza vaccine virus recommendations by the GISRS. The GISAID database has been important to the study of the SARS-CoV-2 as it has the most comprehensive collection of sequences for this virus type. Arguably, its recognition of the rights of specimen and/or data providers have greatly facilitated the sharing of the SARS-CoV-2 genome sequences since January 2020. However, researchers have observed that restriction on re-sharing data could have slowed the pace of research (89). Controlled access to the GISAID database also means that it cannot be directly linked to open databases (e.g., INSDC or GBIF), or otherwise integrated and analyzed more broadly for non-health emergency or biological diversity research (90).

## Animal and Plant Health

Under the CBD, non-human genetic materials that are found within a sovereign state are regarded as its genetic resources, and their collection, management and use may be regulated by the jurisdiction concerned to ensure that there is fair and equitable sharing of benefits arising from the utilization of genetic resources within the jurisdiction concerned. PIC must be obtained from the provider country, mainly through a permit system, and based on MAT. As we have considered above, utilization includes research and development on the genetic or biochemical composition of genetic resources, as well as subsequent applications and commercialization. For instance, a representative sample of an expertly identified organism (known as a voucher) deposited and stored at a facility for authentication and identification falls within the scope of the ABS framework. Certain information that is linked to the voucher, like the name of the person who collected the specimen, the date of collection, the habitat of the collection site and locality of the site, can be part of a valuable database, which may be used for additional research. These vouchers could also be used to build barcoding reference libraries, as they are accompanied by richer and higher quality data. Biobanking arrangements can expand barcoding datasets through the addition of new barcoding markers. Molecular and DNA vouchers help link individual studies with other studies and inferences, and can thereby enhance taxonomic identification. It is unclear if an e-voucher (i.e., a digital object that serves as a voucher), a molecular voucher or a DNA voucher, or otherwise a genetic sequence data also falls within the scope of the ABS framework, although it is argued in this paper that it should be.

Organisms that are of interest to the food and agriculture sectors are referred to as genetic resources for food and agriculture (GRFA), and are—as a global concern—within the purview of the FAO. DSI on GRFA is publicly accessible through appropriately 1,700 online databases, but the number of private DSI on GRFA repositories is unknown. While there is no generally agreed definition for DSI, its application in the context of GRFA has been described in a report commissioned by the FAO to include (on page 5) (91):

“multiple kinds of information usually kept in electronic databases about various biological materials found in GRFA, used to manage GRFA, or to derive value from GRFA. Some…but not all DSI on GRFA is DNA (or RNA) composition information, usually presented as a sequence of nucleotides; is sufficient to synthesize a trait without needing to transfer biological genetic material;…DSI on GRFA that is not DNA or RNA may be essential to identify or synthesize some traits; [or] not require DSI on DNA or RNA to identify or synthesize some traits.”

Although DSI in the context of food security is used in regulations on food safety, product labeling and verifying the identification of food components, repositories include genomic information of species that are not GRFA. Through comparative analysis of the genetic composition of species that are GRFA with those that are not, certain traits of the former may be enhanced or eliminated. For instance, comparative genomic analysis contributed to the enhancement of staple crops like cotton, maize and soybeans with the genetic capability of producing insecticidal proteins (92). Beyond food security and safety, DSI is also an essential component of technologies used for conservation and sustainable use (e.g., DNA barcodes), and in support of research and applications in synthetic biology, as well as to inform non-agricultural applications like drug and vaccine development. The INSDC also serves as a core infrastructure for sharing DSI, other nucleotide sequence data and their metadata in the public domain.

Identical information is maintained by the INSDC at its three constituent nodes [US GenBank, DNA Databank of Japan (DDJB) and the European Molecular Biology Laboratory (EMBL-ENA)] through daily data exchange, with the total size of sequence data exceeding 9 petabytes in 2020 (and even larger if access restricted personal genomic data is included) (93). Databases of the three INSDC nodes comprise annotated sequence, NGS reads, project metadata, biomaterial samples information, functional genomics and human genomic data, and are linked with Accession Numbers that also help to ensure data traceability. Data ownership is retained by data providers, while intellectual property rights are managed independently from INSDC even though patented sequences are deposited within the INSDC by the national or regional patent offices in Europe, Japan, Korea and the US. The data-sharing policy of the INSDC is essentially characterized by the FAIR principles in its practice of free and unrestricted access to all data records, although use restrictions may apply to human genomic data, depending on the informed consent agreements with the donors concerned. Since the outbreak of COVID-19, publicly funded SARS-CoV-2 sequences from the US and the EU are registered with the INSDC and other repositories like GISAID, and INSDC databases may be accessed by researchers without restriction on use or distribution, and without the need to register for access.

It is still moot as to whether DSI falls within the scope of the NP, but if it does, the lower cost and ease of access to high throughput genetic sequencing technology could render the implementation of the ABS framework more demanding. The CBD is directed at regulating the physical transfer of tangible genetic or biological material from the provider country to the user. It does not address the ease of genetically sequencing a species within a provider country and transferring the genetic sequencing data overseas. For instance, devices the size of a credit card for sequencing DNA in saliva or blood samples are being developed to diagnose the most common microbial pathogens in animal GRFA (94). The genetic information or DSI obtained may be easily transmitted to anywhere in the world, and will allow DNA or RNA molecules to be synthesized through biotechnological tools (e.g., gene editing) without the species concerned ever having left the provider country (91). This is similarly the case for pathogens, which in turn raises important ethical and legal concerns, including those that pertains to ABS (95). The German National Academy of Science (Leopoldina) has similarly observed that digitalization in the life sciences and improvements in synthetic biology render traditional forms of biopiracy obsolete since genetic information can be derived locally and imported into globally accessible open databases without the species having to cross any national boundary (90, 96). The sequencing information may have potential or actual commercial value through comparative analysis with other sequences or through the production of encoded protein, without the need to synthesize the entire living organism.

The complexity of the benefit sharing calculus needs little explanation. Maintaining the databases is in reality not without cost; this has been estimated to be around 300 million US dollars annually to maintain the over 1,700 public DSI databases, located mostly in developed countries (91). This cost is likely to be prohibitive to developing countries, without taking into account the added cost of big data infrastructure and related cost in human capital (97). In addition, the INSDC holds more than 1.5 billion sequences and is accessed by 10 to 15 million users a year, and the service it provides is ~50 to 60 million US dollars a year; all cost being met exclusively by the host countries of the three nodes (90).

It is just as complex to evaluate the concept and scope of genetic information that should be delineated as DSI for the purposes of the ABS framework. In a report of the *Ad Hoc* Technical Expert Group (AHTEG) on Digital Sequence Information on Genetic Resources in 2018, it was observed that various types of information may be relevant to the utilization of genetic resources and that “DSI” would be at best a placeholder (other candidate terminologies include “digital genetic resource information”) (98). In a subsequent report, four incremental groups of information have been identified as potentially constituting DSI. Of these, only the first three groups of genetic and biochemical information are regarded as appropriate to describe DSI on genetic resources for the purposes of the ABS framework (99). Group 1 information is closest to the DNA and/or RNA of the species in terms of the degree of biological processing and the proximity of the underlying genetic resources, and will thereby include nucleotide sequence data (NSD) or information, and digital sequence data. Group 2 (which includes Group 1 information and information on proteins and epigenetic modifications) and Group 3 (comprising Group 2 information and information on metabolites and other macromolecules) information include a greater variety of genetic and biochemical information, but could still be defined as DSI for the purposes of the ABS framework (100). However, traditional knowledge associated with genetic resources, information associated with DSI, and other types of information associated with a genetic resource or its utilization is considered too far removed from being classified as “DSI.” Even if so, traditional knowledge of this kind is likely to already be within the remit of the ABS framework.

If DSI falls within the remit of the ABS framework, the extent that access should be regulated through the established mechanisms of PIC and MAT will need to be considered. The ABS-related considerations include: (1) If some form of payment should be required for access; (2) Whether benefit sharing should be linked to a resulting product or service, or simply through the contribution of DSI; (3) If tracing to the country of origin is required; and (4) If benefit sharing should continue to be defined by existing bilateral mechanisms (e.g., permit and contract) or through multilateral mechanisms or arrangements (e.g., global fund) (101–103).

Prominent organizations like the International Chamber of Commerce and the Leopoldina have called for open access to DSI to be maintained as a non-monetary means of global benefit sharing (90, 104). The Leopoldina acknowledges the need to ensure equitable benefit sharing among stakeholder, but has called for a multilateral approach, notably in its proposal of a global fund composed of financial contributions from the Global North and from the profits of commercial entities. It argues strongly against the imposition of financial barriers (e.g., general fee or user fee) to access DSI based on the argument that fairness and equity will not be served since more than half of the users of the INSDC databases that are currently freely available are said to be from the Global South. In contrast, more than half of the sequence information with a known country of origin is from the USA, China, Japan or Canada (105).

The Leopoldina also indicates that legal barriers could hinder information access, with the observation that regulatory and administrative requirements associated with the PIC and MAT requirements under the CBD have burdened or obstructed research. Crucially, a database with restricted access (e.g., the GISAID) cannot be fully linked up with freely and openly accessible databases (e.g., INSDC). A study by Federica Fusi et al. similarly suggests that regulative institutions tend to hinder access, whereas meso-level institutions (e.g., collaborative research establishments) and interpersonal networks tend to promote access by reducing the delays and blockages to accessing biological materials (106). If DSI is to be treated no differently from existing genetic resources that are under the remit of the ABS framework, concerns have been voiced over the economic costs and complexities (particularly administrative ones) that the implicit bilateral arrangements could give rise to. The viability of the INSDC may be brought into question as DSI held by GenBank will not be bound by the ABS framework since the US is not a party to the CDB, whereas the EU and Japan are. Competition among providers of DSI may also arise as a consequence (107). Others have adopted a contrary view, and argued that the inclusion of all DSI in the ABS framework is necessary in response to historical and ongoing exploitative practice, and could better ensure that all stakeholders agree with the terms of data sharing that do not burden Open Science, thereby promoting trust and resource sharing (7).

## Ecosystems and Biodiversity

Data driven science is already generating massive datasets that help to inform the development of biodiversity and environmental indicators (108, 109), and to steer policy and regulation (110). A number of important initiatives on biodiversity have taken root to develop and deploy tools and techniques to generate data and transform them into synthesized information for a range of purposes. The Biodiversity Observation Network of the Group on Earth Observations (GEO BON) has identified 22 Essential Biodiversity Variables (EBVs) to monitor and evaluate biodiversity changes (111), while the Global Biodiversity Information Outlook (GBIO) has put forward 23 goals to develop biodiversity informatics (112). Data is drawn from field surveys and automated sensors, biological collections, molecular data and academic literature. Other data repositories like the Global Biodiversity Information Facility (GBIF) and DataOne have also helped to link up the “long tail of science” by receiving the datasets of small scientific projects. As Claire Lajaunie et al. explain, massive datasets not only enable greater precision in the descriptions of the state of an ecosystem or environment, but could (when supported by integrative modeling) allow different possible trajectories of ecological or environmental changes to be analyzed based on a set of prescribed targets (113). The targets or indicators can themselves be diversified based on stakeholders' or contextual need, thereby also enabling a more holistic assessment through the plurality of development trajectories. However, the choice of appropriate scientific knowledge applied in the indicators and modeling of possible trajectories across a variety of temporal and spatial scales carry ethical implications, including those that relate to fairness and equity. Scenarios that are selected by modelers and the manner in which they are simulated close off other options that may not have been presented or explained to decision-makers and stakeholders (114). The scenarios and/or models may themselves be constrained by limited data, as data integration tends to be limited by different conceptual framings and vocabularies being used.

Ethical caution in guarded reliance on big data analytics and AI-based software in policy and regulatory decision-making does not detract from important benefits that data-driven science could eventually confer. In the meantime, much effort is still needed to improve the overall ecosystem and biodiversity data landscape, and to develop more effective means of data identification and integration. A review by Luiz Gadelha et al. of a selection of biodiversity informatics databases and tools reveals that the use of EBVs as indicators remain challenging due to significant information gaps that prevent global level inferences on the state of biodiversity (115). As a research concern in biodiversity informatics, biological systems modeling has made minimal progress. More granular data on relative abundance of species, their traits and how they interact will be needed to identify trends and make predictions about biodiversity through modeling. Currently, there is still very limited granular data on most organisms, and existing data may contain bias, and may be tainted with taxonomic and geo-referencing errors.

Data collection could be substantially intensified by using AI methods for automating specimen identification, and in this connection, deep learning techniques have been applied for species identification in herbarium sheets (116, 117). For instance, machine learning may also be applied to support open data integration activities, like entity matching (107, 118, 119). While AI methods can help to identify and integrate heterogeneous and dispersed datasets, it is unclear to what extent data providers are incentivized or encouraged to adopt ontologies and metadata standards.

## Discussion

Perhaps more than ever in human history, a diverse pool of data is available to drive the OH strategy as ODH. Data could be molecular (e.g., genomic, serological and metabolic data), observational (e.g., body temperature, weight, feed intake), agronomical (e.g., herd structure, breeding, sanitation), agricultural (or on land use or land cover, more generally), epidemiological, demographical, commercial, meteorological, and so forth. However, data is dispersed in a variety of management systems and may be difficult to locate. Even if data location is not an issue, access may be restricted as it could be owned or controlled by an equally diverse range of individuals or entities (both public and private), and the legal status of the data (e.g., constituting intellectual property or not) itself may not always be clear. In addition to these challenges, there are longstanding concerns that relate to unfair, exploitative and other ethically questionable practices. As we considered in the section above, different views on the costs and other burdens of data management and data sharing are closely associated with fair and equitable dealings with individuals, communities or even countries, whether perceived or real. For sensitive data (e.g., health data), other ethical and regulatory requirements may limit access to purposes indicated by the data subjects concerned. A net result is the inability to draw on data from the different sources and domains (e.g., different levels of living organisms), and across different spatial and temporal scales to develop the AI and related digital capabilities that are needed to operationalize ODH effectively.

The introduction of the FAIR principles in institutional data management could help to address some of the obstacles to data accessibility, although a study found that their explicit use has either been limited or already largely reflected in existing practices (120). More crucial to the discussion in this paper is that the FAIR principles do not provide assurance that data collection, management and use are necessarily consistent with the requirements of fairness and equity or indeed any other ethical principles, or would otherwise lead to ethically acceptable outcomes. Arguably, a multilateral (or global) framework on ODH can help to facilitate data integration by promoting data standardization through setting common data exchange standards to guide sustained data exchange by ecologists, epidemiologists, evolutionists, human and animal healthcare specialists and other knowledge domain professionals (121). Within a collaborative framework, new methodologies (that draw closer together machine learning and statistical modeling) (122) to render different data sources interoperable are more likely to be developed. Data interoperability skills, documentation and metadata are also likely to be better developed and supported within this framework. For instance, the Minimum Information about any Sequence (MIxS) checklist for contextual metadata about biological samples and experimental technologies initiated by the Genomic Standards Consortium illustrates the advantage of operating within a broader framework (93).

Other concerns may also be more effectively addressed at the global level or through multilateral means, including the environmental impact of energy intensive computer resources and equipment (e.g., sensors) that make up data infrastructures around the world, and ABS. In order to promote accountability and trust so that there will be greater willingness to share data and other relevant resources, the global framework on ODH must go beyond the existing ABS framework in at least three different ways. First, it should apply to all genetic resources and data, including DSI, whether from humans or non-humans. Second, the FAIRER principles should be implemented, with focus on fair and equitable benefit-sharing as a crucial component of the “E” or “Ethical” in these principles. Third, the framework should move beyond the bilateral or contractual approach under the current ABS framework, and adopt more multilateral approaches to data sharing (such as the use of federated data systems, discussed below) and to ABS. Whether on its own or in conjunction with the CBD and NP, the ODH framework should augment the ABS framework to one that is ABS+. We discuss each of these features in turn.

### Comprehensive Coverage of Genetic Resources and Data

We have considered above the current debate over the status of DSI in relation to the ABS framework. While there may appear to be advantages, particularly to the scientific community and to the commercial sector, for the current status quo on DSI to be maintained, this is at odds with the current trend in global politics where a growing number of countries are exerting greater control over their biological resources, and by extension, data that is connected with or derived from them. While the Leopoldina takes an “open” database to be one with free and unrestricted access, the AHTEG adopts a more realistic view in its observation that publicly accessible databases do apply different terms and conditions on access and use. In other words, the presence of access restrictions may not burden science if they promote data sharing and trust from a longer term and sustainability point of view. Crucially, both the Leopoldina and the AHTEG recognize the importance of traceability in attributing fair and equitable benefit, and in this regard, three important observations have been made by the AHTEG. First, ease of tracing the country of origin depends on the type of information that could count as DSI, with those in Group 1 being easiest to trace and verify. Second, potential ways of improving traceability include enhancing the inclusion of relevant passport data (e.g., making the provider country a mandatory field entry when the data is uploaded), and more consistent use of the INSDC country tags. Third, machine learning and machine readability of nucleotide sequence listings could help facilitate data consistency between patient information systems and INSDC, and may help to (through interoperability) link internationally recognized certificates of compliance (IRCCs) to genetic sequences uploaded in INSDC (123). While publicly accessible databases may not be as freely accessible as data held by the INSDC, they are not necessarily incompatible with Open Science or Open Research. Control over data access and use could better ensure that equity and other ethical and regulatory requirements are met, and thereby also sustain trust and facilitate greater openness and data exchange. Additionally, data federation (explained below) ensures traceability and can thereby support ABS, as we shall consider below.

### Implementing FAIRER Principles

A study by Jack Heinemann et al. (at page 24) found that failure of legislation to provide a framework for ABS could have the effect of impeding data-sharing as provider jurisdictions may restrict the outflow of genomic data (whether DSI, NDS, or related data forms) through the imposition of compliance or subscription costs. Interested stakeholders (including researchers and commercial entities) may also prioritize the value of the genomic data to the provider jurisdiction or to indigenous populations, and so, either keep the data secret or not collect (or retain) the dataset altogether (91). Citing Pope et al., they observe that researchers have withheld critical information from datasets that are placed in the public domain, thereby compromising its value to subsequent users (124). This is perhaps unsurprising as researchers, even those in high-resource settings (125), report lack of recognition or greater individual costs to publicly share their data (126). The actual and perceived cost of data sharing by researchers in low resource settings is a long standing and as yet unresolved issue. Owing to resource and human capital constraints, DSI databases on GRFA are mainly in developed countries. Although cost of DNA sequencing and synthesis is decreasing, the same cannot be said of the cost in establishing and maintaining digital infrastructure, as well as associated specialist training that is required. INSDC is supported by the host governments of the three nodes, although governance is independent of any political or scientific body and based on the FAIR principles. Even if data held by the INSDC is freely accessible, this does not detract from the fact that ultimate control over these important databases are in the hands of the wealthiest countries in the world.

As we have considered, the CBD and NP seek to ensure fair and equitable sharing of benefits that arise from the utilization of genetic resources for the purposes of advancing the goals of conservation of biological diversity and sustainable development. In this context, benefits may be monetary or non-monetary, and could take a number of forms, like technological development, access to new medicines that address the health needs of local populations, knowledge exchange and support for local projects or initiatives (127). Fair and equitable ABS under ODH (or ABS+) will need to be broader because it is concern with data that either does not fall within the CBD and NP regimes (e.g., human behavioral data), or not clearly so (e.g., DSI). In other words, the FAIRER principles under ABS+ will need to be more closely aligned with global justice that underscores the SDGs. Global justice is not only concerned with transactional fairness, but seeks to address deep seated structural inequities that may be within particularly jurisdictions or in the international order (128). In the context of ODH, a question may be asked as to the extent that countries are equally capable of developing data driven solutions in response to ever growing challenges in the human-animal-ecosystem interface. As a practical matter, it may also be asked what structural arrangements that ODH as the feature of a just global order could adopted to facilitate fair and equitable assemblage and sharing of data. Charles Lawson et al. have proposed an approach that to shift the handling of information (like DSI) into the ABS domain in order to ensure fair and equitable ABS (129). I propose scaling up the federated data approach, which is being implemented within (rather than across) specific data domains (e.g., purely human health-related research).

Consider human genomics research, where data is relatively homogenous (i.e., just one species) and where there has been a history of support from important funding agencies for open sharing of data through shared repositories and other means, formidable impediments to data sharing remain (78). The vision of free and open access (similar to access via the INSDC) to genome data encapsulated in the Bermuda Principles that were formulated for the Human Genome Project remains obscured by ethical, legal and social concerns (130), notably for the lack of diversity in genome databases where African people and indigenous populations are not well represented, disproportionate focus on the study of diseases that tend to be more prevalent in high income countries, and limited contribution to capacity building in developing countries, whether in terms of computational infrastructure or good data governance practices (131). And where personal (e.g., health and genomics) data is concerned, we have also noted that a growing number of countries have introduced laws to protect personal data privacy and security. In contrast to the “all-or-nothing” approach (whereby data is either open and freely accessible or not), a tiered access approach through federated data systems may be the best way forward in terms of balancing data needs with ethical and regulatory requirements. By this approach, data need not be consolidated into one repository, but may be distributed across different databases and in a variety of repositories around the world (8). These databases are virtually connected through a software interface (known as application programming interface or API), which sets out a defined protocol that enables the different institutional data management systems to communicate with one another and to exchange information. Such a federated data system allows authorized users to retrieve data from a federated network of organizations, but without the need for the relevant datasets to be transferred out of any of these organizations. Where the sharing of human genomics and health data is concerned, the Global Alliance for Genomics and Health has developed a federated data ecosystem to enable responsible and effective data sharing (79). The use of APIs allows the definition and enforcement of specific governance policies (encompassing ethical and legal requirements), and could thereby balance control with equity and other ethical concerns in a manner that is envisaged in the ABS Framework. This arrangement is also closer to the FAIRER principles, precisely because equity and other ethical considerations may be actively and explicitly engaged with (132).

Federated data sharing models are not limited to human genomics and health data either. About a decade ago, Reichman et al. surmised the three major technical challenges to more open sharing of data as data dispersion, heterogeneity and provenance (80). The establishment of well-curated, federated depositories (like DataOne) and the use of structured metadata specifications have helped to address these challenges (133). AI approaches could also enhance the effectiveness of data federation by supporting greater participation (e.g., the use of AI expert systems), allowing more precise and dynamic targeting of data to be collected and avoiding the collection of reductant (collinear) data (134, 135). While AI systems may themselves be complex and difficult for laypersons to use, there are now citizen science projects where communities could be involved in the design and construction of AI tools (136). Knowledge representation methods from symbolic AI (such as the use of domain specific language (DSL) in advanced software engineering) helps to add transparency and reproducibility, by making model components accessible in readable structured text file rather than computer codes (137). Studies in healthcare and conservation shows that uptake of digital technologies needs to be carefully orchestrated, rather than adopted as a matter of course, and is likely to be most effective when it is participatory. For instance, Tara Slough et al. find that relying only on deforestation alerts generated by remote-sensors from monitoring tree cover loss in the Amazon is more effective when coupled with a community monitoring program where populations living on the deforestation frontiers are trained and incentivized to patrol communal forests while provided with access to early deforestation alerts (138, 139). The more participatory approach that AI systems enables reinforces fair and equitable associations, and so ensures that the human-animal-ecosystem interfaces are drawn closer together not only at a technical level, but also in terms of shared goals for collective action.

### Adopt Multilateralism

The International Treaty on Plant Genetic Resources for Food and Agriculture (ITPGR) has been proposed as a possible model that could support a multilateral approach for ABS. Genetic resources in relation to 35 food crops and 29 forage genera of global importance shared under the ITPGR are exempted from ABS Framework as it consist of a specialized international instrument for ABS, unless access is restricted through intellectual property rights (140). A view put forward is to, like the ITPGR, carve out exceptions to the ABS Framework for biodiversity research. This could then ensure greater accessibility of genetic resources and related data, like microbial culture collections held by the World Federation of Culture Collections, taxonomic type materials held by museums globally (77). As biodiversity research is only one domain of ODH, such a proposed exemption will not operationalize ODH which needs to draw data across all three domains, and could even result in greater data fragmentation. There are however advantages to shifting away from bilateral contractual arrangements in the ABS Framework and toward greater multilateralism, as observed by the WHO in its review of the relationship between the PIP Framework and the NP discussed earlier in this paper (86, 90). As noted above, data federation could be a step in this direction, and may help to avoid current problems with onerous administrative requirements, procedures that are unclear or lacking in transparency, and seemingly endless bureaucracy. Federated data systems developed under the proposed ABS+ framework could allow valorisation of data at a macro or system level, rather than only at the level where data is be held or controlled, and optimize calculation performance in real time (29). Such an approach would better serve the needs of ODH as an initiative under the SDGs.

## Conclusion

Growing climate exigency and related threats to human, animal and plant health makes the operationalization of OH more pressing than ever. The digitalization and operationalization of OH as ODH is a means to deploy digital technologies (including AI and big data) to better capacitate us to deal with these challenges through procuring better and more detailed data across different knowledge domains to gain new and deeper insights, make better predictions and plans, monitor and evaluate changes—especially those pertaining to the emergence or reemergence of infectious diseases—and implement preventive strategies and public health countermeasures. However, the data landscape is fragmented and access to certain types of data is likely to become more restrictive as individuals, communities and countries seek to assert greater control over data owing to a number of concerns, including unfair access to benefits that may arise, whether directly or indirectly, from data taken from them. From a global perspective, the ABS framework narrowly addresses this concern for non-human biological materials and related data, but it suffers from a number of limitations, including its reliance on bilateral arrangements between contracting parties, and potentially high transaction costs attached. It also does not deal explicitly with data collection and use, and consequently, it is unclear if certain types of data like DSI fall within its remit.

This paper argues for a dedicated global ODH framework that also augments current ABS provisions in the CBD and NP as an ABS+ Framework. In essence, this ODH framework should apply to both human and non-human biological materials and related data, put into effect the FAIRER principles (more aligned with global justice under the SDGs) through arrangements like federated data systems, and shift toward multilateralism. By operationalizing OH through digitalization and the deployment of AI and other digital capabilities under the proposed ODH Framework, we are more likely to be able to protect and restore natural habitats, secure the health and well-being of all living things, and thereby realize the goals set out in the post-2020 Global Biodiversity Framework under the CBD (141).

## Data Availability Statement

The original contributions presented in the study are included in the article/supplementary material, further inquiries can be directed to the corresponding author/s.

## Author Contributions

The author confirms being the sole contributor of this work and has approved it for publication.

## Funding

This work was supported by a grant on health care digitalization from the WYNG Foundation, Hong Kong SAR.

## Conflict of Interest

The author declares that the research was conducted in the absence of any commercial or financial relationships that could be construed as a potential conflict of interest.

## Publisher's Note

All claims expressed in this article are solely those of the authors and do not necessarily represent those of their affiliated organizations, or those of the publisher, the editors and the reviewers. Any product that may be evaluated in this article, or claim that may be made by its manufacturer, is not guaranteed or endorsed by the publisher.
